# Supporting implementation of new anti-tuberculosis regimens: the role of Brazil in the FAST-TB End-User Survey

**DOI:** 10.36416/1806-3756/e20260343

**Published:** 2026-09-14

**Authors:** Denise Rossato Silva, Fernanda Carvalho de Queiroz Mello, Tatiana Galvão, Rosella Centis, Lia D’Ambrosio, Alessandra Bellavia, Giovanni Battista Migliori

**Affiliations:** 1. Faculdade de Medicina, Universidade Federal do Rio Grande do Sul - UFRGS - Porto Alegre (RS) Brasil.; 2. Instituto de Doenças do Tórax, Universidade Federal do Rio de Janeiro - UFRJ - Rio de Janeiro (RJ) Brasil.; 3. Universidade Federal da Bahia, Salvador (BA) Brasil.; 4. Servizio di Epidemiologia Clinica delle Malattie Respiratorie, Istituti Clinici Scientifici Maugeri - IRCCS - Tradate, Italia.; 5. Public Health Consulting Group, Lugano, Switzerland.; 6. Dipartimento di Promozione della Salute, Materno-Infantile, di Medicina Interna e Specialistica di Eccellenza, Unità Operativa Complessa di Astanteria, Medicina e Chirurgia di Accettazione e Urgenza - MCAU - Azienda Ospedaliera Universitaria Policlinico ‘Paolo Giaccone’ Università degli Studi di Palermo, Palermo, Italia.

Tuberculosis remains a major global public health problem, with millions of cases and over a million deaths annually.^1^ Although new, shorter, more effective treatment regimens are being developed, their adoption by national tuberculosis programs is often slow due to issues such as financial constraints and regulatory processes.^2^ Therefore, understanding the needs and expectations of end users-people affected by tuberculosis, health care workers, researchers, managers, and civil society organizations-is essential to identify bottlenecks and accelerate equitable adoption of new treatment regimens.^3^

Facilitating Accelerated Science and Translation for TB Regimen Development (FAST-TB), a project supported by the US National Institute of Allergy and Infectious Diseases in collaboration with the Civilian Research and Development Foundation, has an arm designated Research to Translate and Yield Evidence into Practice, which was established to provide a comprehensive response to improve the adoption and rapid implementation of new tuberculosis treatment regimens.^4^ To that end, a global survey involving more than 1,300 participants, designated the FAST-TB End-User Survey, was conducted, complemented by in-depth interviews in a large multinational sample with representatives from various stakeholder groups, including more than 500 members of the affected communities and the front-line health care workers. The questionnaire was made available in six languages and covered 137 countries, including all eight countries with the highest tuberculosis burden, representing more than 80% of the cases worldwide.^3^ In addition, a two-round international Delphi consensus process was employed to identify priorities and develop recommendations for future research and implementation strategies. The final list of priorities was organized into three areas: treatment characteristics and user preferences for drug-susceptible tuberculosis, drug-resistant tuberculosis, and tuberculosis preventive treatment-including long-acting injectables-with a focus on efficacy, duration, and safety; implementation and scale-up of interventions, in which operational challenges, training, logistics, and regulation are taken into consideration; and access and sustainability, for which barriers to early diagnosis, financing, and equitable access are addressed. For long-acting injectables, acceptability depends not only on the characteristics of the injections but also on the injection site and the need for monitoring, as well as privacy, stigma, and trust in health care services.^5^

Brazil plays a key role in the FAST-TB End-User Survey. The project as a whole was approved by the Research Ethics Committee of the Hospital de Clínicas de Porto Alegre (Reference no. 90411725.3.0000.5327), where the data collected with the Research Electronic Data Capture tool are hosted. Researchers in Brazil are also contributing across the three core components: participating in the expert panel; collecting questionnaire responses; and participating in the health care worker interviews.

In conclusion, integrating survey responses with the output of patient and health care worker interviews will provide critical evidence to address the priority research questions identified through the Delphi process and inform the design of more acceptable, feasible, and implementation-ready clinical trials of tuberculosis treatments in the coming decade.
